# Proteomic Analysis Highlights the Impact of the Sphingolipid Metabolizing Enzyme β-Galactosylceramidase on Mitochondrial Plasticity in Human Melanoma

**DOI:** 10.3390/ijms25053062

**Published:** 2024-03-06

**Authors:** Davide Capoferri, Luca Mignani, Marcello Manfredi, Marco Presta

**Affiliations:** 1 Department of Molecular and Translational Medicine, University of Brescia, 25123 Brescia, Italy; davide.capoferri@unibs.it (D.C.); luca.mignani1@unibs.it (L.M.); 2 Department of Translational Medicine, University of Piemonte Orientale, 13100 Novara, Italy; marcello.manfredi@uniupo.it; 3 Consorzio Interuniversitario Biotecnologie (CIB), Unit of Brescia, 25123 Brescia, Italy

**Keywords:** melanoma, proteomics, sphingolipids, β-galactosylceramidase, mitochondrion

## Abstract

Mitochondrial plasticity, marked by a dynamism between glycolysis and oxidative phosphorylation due to adaptation to genetic and microenvironmental alterations, represents a characteristic feature of melanoma progression. Sphingolipids play a significant role in various aspects of cancer cell biology, including metabolic reprogramming. Previous observations have shown that the lysosomal sphingolipid-metabolizing enzyme β-galactosylceramidase (GALC) exerts pro-oncogenic functions in melanoma. Here, mining the cBioPortal for a Cancer Genomics data base identified the top 200 nuclear-encoded genes whose expression is negatively correlated with *GALC* expression in human melanoma. Their categorization indicated a significant enrichment in Gene Ontology terms and KEGG pathways related to mitochondrial proteins and function. In parallel, proteomic analysis by LC-MS/MS of two *GALC* overexpressing human melanoma cell lines identified 98 downregulated proteins when compared to control mock cells. Such downregulation was confirmed at a transcriptional level by a Gene Set Enrichment Analysis of the genome-wide expression profiling data obtained from the same cells. Among the *GALC* downregulated proteins, we identified a cluster of 42 proteins significantly associated with GO and KEGG categorizations related to mitochondrion and energetic metabolism. Overall, our data indicate that changes in *GALC* expression may exert a significant impact on mitochondrial plasticity in human melanoma cells.

## 1. Introduction

Usually resistant to chemotherapy and radiotherapy, metastatic melanoma represents the deadliest form of skin cancer [1,2]. Numerous observations have shown that metabolic reprogramming drives melanoma progression and metastasis, both characterized by significant changes in energetic metabolism. Indeed, melanoma displays a heterogeneous dynamism between glycolysis and oxidative phosphorylation due to tumor adaptation to genetic and microenvironmental alterations [3]. For instance, the tumor driving BRAF^(V600E)^ mutation, which is present in approximately 50% of human melanomas [4,5], suppresses mitochondrial oxidative phosphorylation and drives aerobic glycolysis through the activation of hypoxia inducible factor 1 subunit alpha [6,7]. From a therapeutic perspective, mitochondrial plasticity may confer resistance to targeted therapies in melanoma, including immunotherapy [8].

In this frame, experimental evidence indicates that various sphingolipids, including ceramide, exert a key role in metabolic reprogramming by affecting mitochondrial dynamics, cellular bioenergetics, apoptosis, and mitophagy [9]. Thus, a better understanding of the impact of an altered expression of sphingolipid-metabolizing enzymes on mitochondrial plasticity may provide novel insights about their contribution to melanoma progression and for the development of therapeutic strategies targeting mitochondrial dynamics [10].

The lysosomal acid hydrolase β-galactosylceramidase (GALC; EC 3.2.1.46) catalyzes the cleavage of β-galactose from β-galactosylceramide and other sphingolipids [11,12]. Observations in our laboratory indicate that GALC might function as an oncogenic enzyme in human melanoma. Indeed, the progression from common nevi to stage IV melanoma is accompanied by a progressive increase of *GALC* expression in human skin specimens as assessed by mRNA in situ hybridization [13]. In addition, *Galc* knock-down causes a significant alteration of the lipidomic profile of murine melanoma B16 cells hampering their tumorigenic and metastatic activity. In keeping with these observations, *GALC*-silenced human melanoma A2058 cells were characterized by a decrease in their tumorigenic potential [13].

Here, in an attempt to gain further insights into the pro-oncogenic role of GALC in human melanoma, we performed the categorization of the top 200 nuclear-encoded genes whose expression is negatively correlated with *GALC* expression in the TCGA Skin Cutaneous Melanoma data set using the cBioPortal for Cancer Genomics platform [14]. The analysis of this list of genes on different platforms identified various enriched Gene Ontology (GO) terms and Kyoto Encyclopedia of Genes and Genomes (KEGG) pathways related to mitochondrial proteins and function. Accordingly, by taking advantage of previous proteomic data obtained in our laboratory by liquid chromatography–tandem mass spectrometry (LC-MS/MS) analysis [15,16], we identified a set of 98 proteins whose expression is downregulated at protein and Mrna levels in *GALC* overexpressing A2058 and A375 human melanoma cells (upGALC cells) harboring the BRAF^(V600E)^ mutation. Among them, a STRING-defined cluster of 42 downregulated proteins was associated with GO and KEGG categorizations related to mitochondrion and energetic metabolism. Overall, our data indicate that *GALC* upregulation may exert a significant impact on mitochondrial plasticity in human melanoma cells.

## 2. Results

### 2.1. Negative Correlation between GALC and Nuclear-Encoded Mitochondrial Gene Expression in Human Melanoma

**Figure 1:**
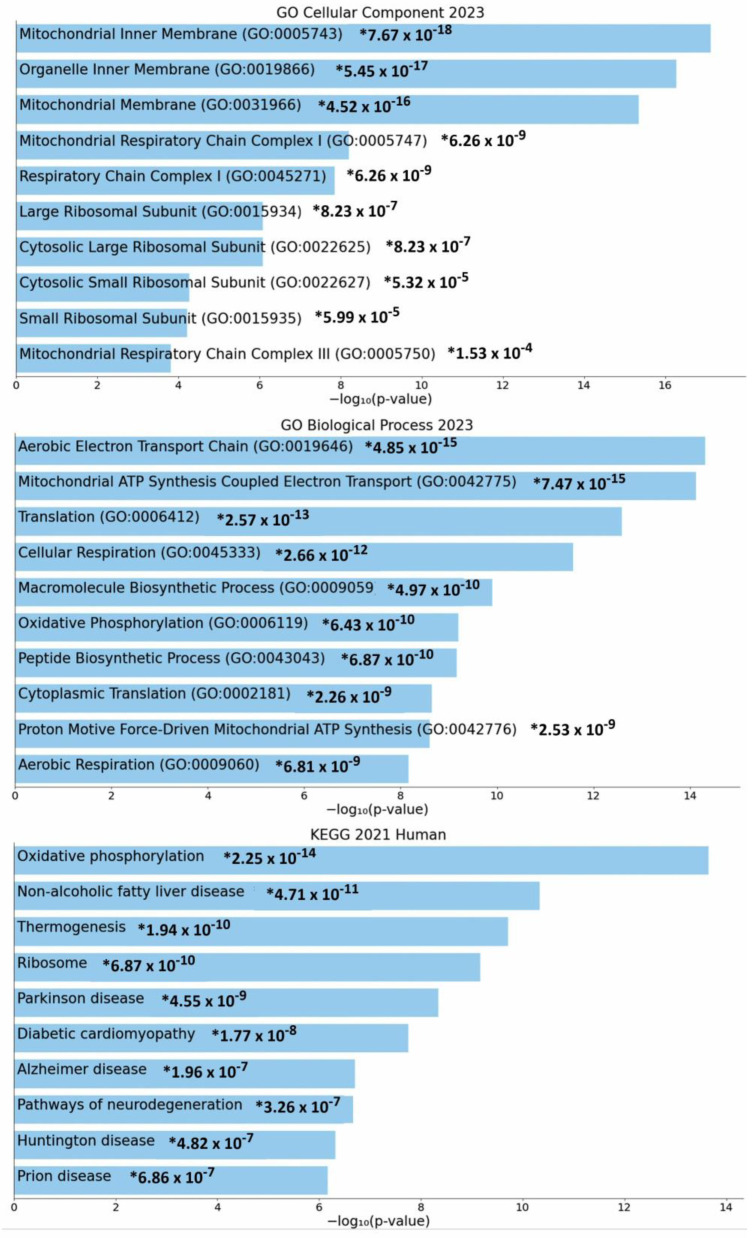
Gene Ontology and KEGG categorization of the top 200 genes whose expression levels are negatively correlated with *GALC* expression in human melanoma specimens following data mining on the cBioPortal for Cancer Genomics platform.

**Figure 2:**
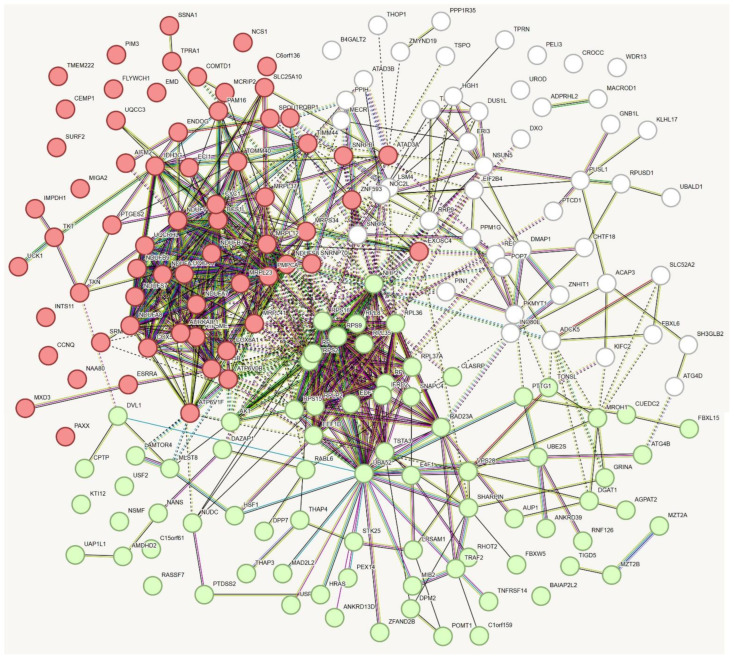
STRING analysis of the top 200 genes whose expression levels are negatively correlated with *GALC* expression in human melanoma following data mining on the cBioPortal for Cancer Genomics platform. The two clusters are defined by the GO terms “Oxidative phosphorylation” (in red) and “Structural constituent of ribosome” (in green).

Data mining was performed on the cBioPortal for Cancer Genomics platform (TCGA Skin Cutaneous Melanoma, Firehose legacy, PanCancer Atlas) to identify those genes whose expression was negatively correlated with *GALC* expression in 472 human skin melanoma specimens. The top 200 genes (Supplementary Table S1) were selected and analyzed using the Enrichr tool (https://maayanlab.cloud/Enrichr/, accessed on 11 January 2024). As shown in Figure 1, the categorization of the selected genes identified various enriched GO Cellular Component and Biological Process terms related to mitochondrial proteins and function, including, among others, “Mitochondrial inner membrane” (*p* value = 7.7 × 10^−18^) and “Aerobic electron transport chain” (*p* value = 4.9 × 10^−15^). Accordingly, “Oxidative phosphorylation” was the top enriched KEGG pathway (*p* value = 2.2 × 10^−14^). In keeping with these observations, STRING analysis identified two major clusters (k-means clustering, Protein–Protein Interaction enrichment *p* value ≤ 1.0 × 10^−16^) of 70 and 62 nodes, defined by the enriched GO terms “Oxidative phosphorylation” (FDR = 3.2 × 10^−16^) and “Structural constituent of ribosome” (FDR = 1.73 × 10^−9^), respectively (Figure 2).

**Figure 3:**
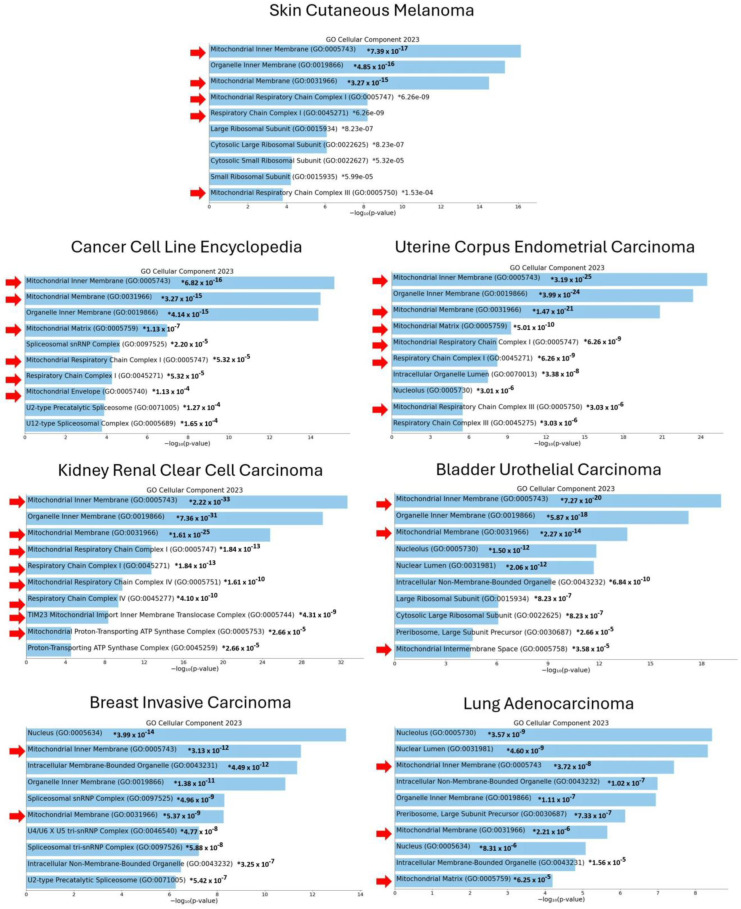
GO categorization of the genes negatively correlated to *GALC* expression in human cancers. GO categorization was performed on the top 200 genes whose expression levels are negatively correlated with *GALC* expression in tumor cell lines (Cancer Cell Line Encyclopedia) and human tumors (TCGA, Firehose Legacy) following data mining on the cBioPortal for Cancer Genomics platform. Arrows highlight enriched GO Cellular Component terms related to mitochondrial structure and function.

Of note, similar results were obtained when GO categorization analysis was performed on the top 200 genes identified on the cBioPortal for Cancer Genomics platform whose expression was negatively correlated with *GALC* mRNA levels in other tumor types, including endometrial carcinoma, renal clear cell carcinoma, urothelial carcinoma, breast invasive carcinoma, and lung adenocarcinoma (TCGA, Firehose Legacy), as well as in the 1736 cell lines included in the Cancer Cell Line Encyclopedia (Supplementary Table S2 and Figure 3). Together, these data suggest that a relationship may exist between *GALC* expression and mitochondrial function in various human cancers, including melanoma.

### 2.2. Proteomic Analysis of Downregulated Proteins in GALC-Overexpressing Melanoma Cells

To address the possibility that a relationship may exist between *GALC* expression and mitochondrial function in human melanoma, we took advantage of previous experiments performed in our laboratory [15,16], in which we investigated the proteomic profile of the cell extracts of *GALC*-overexpressing A2058 and A375 human melanoma cell lines harboring the tumor-driving BRAF^(V600E)^ mutation, which is present in approximately 50% of human melanomas [4,5]. A hierarchic analysis performed by comparing the A2058-upGALC *plus* A375-upGALC protein data sets to the A2058-mock *plus* A375-mock data sets indicated that 304 and 340 proteins are up- or down-regulated (Q value < 0.05) in upGALC vs. mock cells [16]. The categorization of these proteomic data indicates that GALC exerts a significant impact on the proteomic landscape of these cells, leading to the modulation of the expression of proteins involved in various aspects of melanoma progression, including endoplasmic reticulum responses, metastasis, and immune escape.

**Figure 4:**
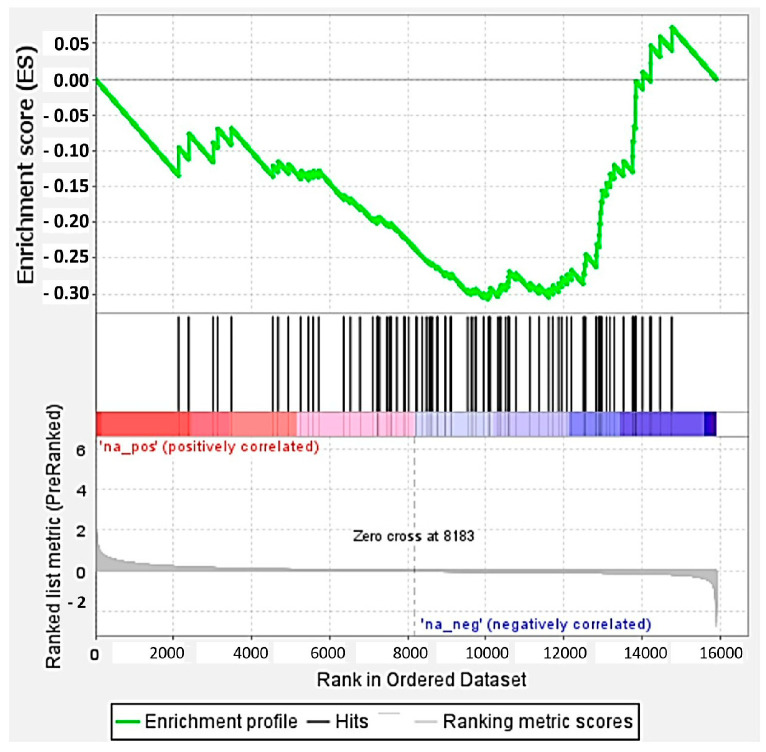
GSEA of GEP data from *GALC*-overexpressing melanoma cells. The expression levels of the gene encoding for the 98 proteins downregulated in A258-upGALC and A375-upGALC vs. mock cells were calculated from GEP data by GSEA.

Starting from these results, we decided to refine this analysis by focusing on the 98 proteins whose amount was reduced by more than 33% in *GALC*-overexpressing cells (corresponding to a fold change < 0.67 in upGALC cells when compared to mock cells) (Supplementary Table S3). This cutoff is based on the observation that a decrease in protein levels lower than 30% is usually devoid of a significant phenotype in heterozygous carriers of genetic diseases. Of note, Gene Set Enrichment Analysis (GSEA) of the genome-wide expression profiling (GEP) data obtained from A2058-upGALC and A375-upGALC cells versus mock cells indicated that the decrease in the amount of the 98 proteins caused by *GALC* overexpression is accompanied by the transcriptional downregulation of the corresponding genes (Figure 4).

**Figure 5:**
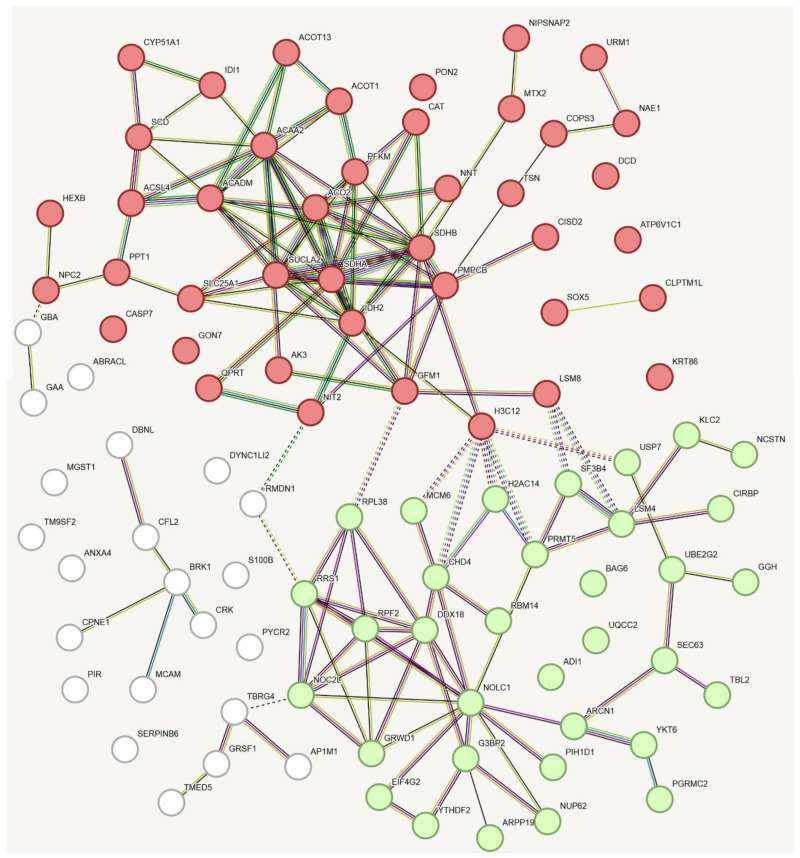
STRING analysis of the proteins downregulated in upGALC melanoma cells when compared to mock cells. The two clusters are defined by the GO terms “TCA cycle” and “Mitochondrion” (in red) and “Ribonucleoprotein complex biogenesis” and “RNA binding” (in green).

In keeping with what was observed for the top 200 genes whose expression was negatively correlated with *GALC* mRNA levels in human melanoma specimens, the categorization of the 98 proteins downregulated in upGALC cells indicated that “Citrate (TCA) cycle” was the most enriched KEGG pathway (*p* value = 3.3 × 10^−7^). Accordingly, “Mitochondrial matrix” (*p* value = 1.1 × 10^−6^) and “Mitochondrial membrane” (*p* value = 6.2 × 10^−6^) were highly enriched GO Cellular Component terms as assessed on the Enricher platform. Again, STRING k-means clustering analysis identified two major clusters in the data set of the *GALC* downregulated proteins (Protein–Protein Interaction enrichment *p* value ≤ 1.0 × 10^−16^) (Figure 5). One “ribosome-related” cluster was formed by 34 terms and was characterized by the GO Biological Process and Molecular Function terms “Ribonucleoprotein complex biogenesis” and “RNA binding” (FDR = 2.7 × 10^−6^ and 1.8 × 10^−6^, respectively). The second “mitochondrion-related” cluster of 42 nodes was defined by the enriched GO Biological Process and Molecular Function terms “TCA cycle” and “Mitochondrion” (FDR = 9.3 × 10^−7^ and 7.1 × 10^−7^, respectively). The list of these proteins with a brief description of their biological function is shown in Table 1. Together, these data indicate that GALC upregulation exerts a significant impact on mitochondrial plasticity in human melanoma cells.

## 3. Discussion

Alterations in the metabolism of sphingolipids, including the tumor suppressor ceramide, exert a deep impact on melanoma [57,58,59,60]. GALC is a lysosomal sphingolipid-metabolizing enzyme that catalyzes the removal of galactose from terminal β-galactose-containing sphingolipids, including β-galactosylceramide [11,12]. Previous observations have shown that GALC may exert pro-oncogenic functions in human melanoma [13,15]. The present work extends these observations and indicates that GALC exerts a significant impact on melanoma mitochondrial plasticity.

Data mining performed on the cBioPortal for Cancer Genomics platform indicated that GO terms related to endoplasmic reticulum/Golgi cellular components are overrepresented among the top 25 genes whose expression is positively correlated with *GALC* transcript levels in the human TCGA Skin Cutaneous Melanoma data set. At variance, the expression of other lysosomal sphingolipid-metabolizing enzymes was associated with gene sets enriched in lysosome-related GO terms [61]. These observations indicated that *GALC* might be selectively involved in the upregulation of endoplasmic reticulum/Golgi intracellular pathways affecting melanoma progression, such as the autophagy and stress of the endoplasmic reticulum [62].

Here, in an attempt to gain further insights into the pro-oncogenic role of GALC in human melanoma, we performed the categorization of the top 200 nuclear-encoded genes whose expression is negatively correlated with *GALC* expression in the same TCGA Skin Cutaneous Melanoma data set. The categorization of the selected genes identified various enriched GO terms related to mitochondrial proteins and function, such as “Mitochondrial inner membrane” and “Aerobic electron transport chain”. Accordingly, “Oxidative phosphorylation” represented the top enriched KEGG pathway and a STRING analysis confirmed the presence of a major cluster of genes negatively correlated with *GALC* expression defined by the enriched GO term “Oxidative phosphorylation”.

The GO categorization analysis of the top 200 genes whose expression is negatively correlated with *GALC* expression confirmed the enrichment of GO terms related to mitochondrial plasticity, also for the endometrial carcinoma, renal clear cell carcinoma, urothelial carcinoma, breast invasive carcinoma, and lung adenocarcinoma TCGA data sets, as well as for the tumor cell lines included in the Cancer Cell Line Encyclopedia. In this frame, *ATG4D*, *ATAD3A*, and *MRPL41* were the top three genes negatively correlated with *GALC* expression in the TCGA Skin Cutaneous Melanoma database. They encode for autophagy-related 4D cysteine peptidase, ATPase family AAA domain containing 3A, and mitochondrial ribosomal protein L41, all spatially located in the mitochondrial matrix and associated with autophagy and mitophagy processes [63,64,65]. Still, when sorted by statistical significance, *ATG4D*, *ATAD3A*, and *MRPL41* are found in less apical positions in the lists of the top 200 genes negatively correlated with *GALC* expression in the other human cancer data sets investigated here (see Table S2). In addition, no relationship occurred between *GALC* expression and mitochondrial plasticity when GO categorization was performed on the TCGA data sets of lung squamous cell carcinoma, head and neck squamous cell carcinoma, and esophageal carcinoma (Supplementary Figure S1). Thus, a contextual relationship appears to exist between *GALC* expression and mitochondrial plasticity in different human cancers.

Such relationship was confirmed by the analysis of the proteomic data obtained by LC-MS/MS on human melanoma A2058 and A375 cell lines that had been engineered to stably overexpress human *GALC* by lentiviral infection [15]. *GALC* overexpression results in an increased tumorigenic potential in these cells and in significant changes in their proteomic landscape, leading to the modulation of the expression of proteins involved in various aspects of melanoma progression, including endoplasmic reticulum responses, metastasis, and immune escape [15,16]. Here, we focused our attention on a set of 98 proteins whose cellular levels were significantly downregulated in *GALC*-overexpressing cells when compared to control cells. These proteins were characterized by the enrichment of the GO terms “Mitochondrial matrix” and “Mitochondrial membrane”, and by the “Citrate (TCA) cycle” KEGG pathway. Such downregulation was confirmed at a transcriptional level by GSEA of the GEP data obtained from the same cells. Among these downregulated proteins, we identified a STRING cluster of 42 proteins significantly associated with the GO terms “Mitochondrion” and “TCA cycle”. Among them, 21 proteins showed a subcellular mitochondrial localization, 6 proteins were associated with mitochondrial fatty acid metabolism, and 8 proteins were associated with the TCA cycle (see Table 1 for details). It is worth noticing that, at variance with what we found in the TCGA Skin Cutaneous Melanoma database, the *ATG4D*, *ATAD3A*, and *MRPL41* encoded proteins do not appear to be significantly downmodulated in our *GALC*-overexpressing melanoma cells, enforcing the concept that the effect of GALC on mitochondrial plasticity may be context-dependent and related to the genetic heterogeneity that characterizes human tumors, including melanoma.

Mitochondrial plasticity plays a pivotal role in different aspects of melanoma biology [6,7,8]. The present data indicate that further studies will be required to assess whether a cause–effect relationship may exist between the mitochondrial protein changes induced by *GALC* overexpression in melanoma cells and its impact on other features of melanoma progression highlighted by our previous observations [62], including endoplasmic reticulum responses, autophagy, metastasis, and immune escape.

Human melanoma A2058 and A375 cells harbor the tumor-driving BRAF^(V600E)^ mutation, which is present in approximately 50% of human melanomas [4,5] and represents a major target in melanoma therapy [66]. The BRAF^(V600E)^ mutation has been shown to suppresses mitochondrial oxidative phosphorylation and to drive aerobic glycolysis through the activation of hypoxia inducible factor 1 subunit alpha [6,7]. Our data indicate that *GALC* upregulation can exert a further impact on mitochondrial plasticity in a *BRAF* mutated background. This might be associated with modifications in the sphingolipid landscape consequent to the increased GALC enzymatic activity in melanoma cells. Indeed, sphingolipids exert a key role in metabolic reprogramming by affecting mitochondrial dynamics, cellular bioenergetics, apoptosis, and mitophagy. Of note, ceramide can affect the activity of the mitochondrial electron transfer chain with inhibitory effects on mitochondrial Complex I and Complex IV and can cause a decrease in mitochondrial membrane potential and ATP depletion, its mitochondrial accumulation leading to an increase in ROS production (see [9] and references therein).

Mitochondrial plasticity may confer resistance to targeted therapies in melanoma, including immunotherapy [8]. Understanding the role of sphingolipids and sphingolipid-metabolizing enzymes on mitochondrial dynamics may provide novel information for the development of efficacious approaches in mitochondrial targeting cancer therapies [10].

## 4. Materials and Methods

### 4.1. cBioPortal Data Mining

The cBioPortal for Cancer Genomics platform (TCGA Skin Cutaneous Melanoma, Firehose legacy, PanCancer Atlas) was used to identify those genes whose mRNA levels were negatively correlated with *GALC* expression in human skin melanoma (n = 472). The identified genes were ranked according to the *p* value of their negative correlation, and the top 200 genes were selected. The Enrichr tool (https://maayanlab.cloud/Enrichr/ accessed on 11 January 2024) was used to perform their categorization by Gene Ontology [67,68] (GO Cellular Component and Biological Process 2023 Ontologies) and Kyoto Encyclopedia of Genes and Genomes [69] (KEGG 2021 Human Pathways). In addition, the identified genes were clustered by k-means clustering (n = 3) on the online STRING platform (https://string-db.org, version 12.0, accessed on 11 January 2024) [70].

GO categorization analysis was performed on the cBioPortal for Cancer Genomics platform also on the top 200 genes negatively correlated to *GALC* mRNA levels in the TCGA Firehose Legacy data sets of human endometrial carcinoma (n = 549), renal clear cell carcinoma (n = 538), urothelial carcinoma (n = 413), breast invasive carcinoma (n = 1108), lung adenocarcinoma (n = 586), lung squamous cell carcinoma (n = 511), head and neck squamous cell carcinoma (n = 530), and esophageal carcinoma (n = 186), as well as in the Cancer Cell Line Encyclopedia (n = 1739).

### 4.2. Proteomic Analysis

GALC-overexpressing A2058-upGALC and A375-upGALC melanoma cells and the corresponding control mock cells have been described in a previous publication [15]. In this present work, we investigated the proteomic data obtained by LC-MS/MS analysis of the extracts of the A2058 and A357 mock and GALC-overexpressing cells [16]. Peak intensity values of the identified proteins were first transformed to a log scale (plus 1 to avoid zero values), and Q values < 5% were considered to identify differentially expressed proteins due to their high statistical power.

### 4.3. Categorization of Proteomic Data

Proteins identified by LC-MS/MS analysis with a fold change lower than 0.67 were clustered by k-means clustering (*n* = 3) on the STRING platform, whereas protein categorization was performed for GO Cellular Component and Molecular Function 2023 ontologies and KEGG 2021 Human pathways using the Enrichr platform.

### 4.4. Gene Set Enrichment Analysis

GSEA [71,72] was run on the genome-wide expression profiling (GEP) data obtained from the total RNA extracted from A2058-upGALC and A375-upGALC melanoma cells and from the corresponding control mock cells according to standard procedures.

## Figures and Tables

**Table 1:** List of the proteins down-regulated upon *GALC* transduction in human melanoma cells belonging to the “mitochondrion-related” STRING cluster shown in red in Figure 3. Each entry is completed by the name of the gene encoding for the listed protein and by a brief description of its biological function in cancer, including melanoma when available.

Protein	Gene	Biological Function
Acetyl-CoA Acyltransferase 2	*ACAA2*	3-ketoacyl-CoA thiolase, mitochondrial. Catalyzes the last step of the mitochondrial beta-oxidation pathway. Its activity promotes melanoma cell survival and metastasis [17]
Acyl-CoA Dehydrogenase Medium Chain	*ACADM*	Medium-chain specific acyl-CoA dehydrogenase, mitochondrial. Catalyzes the initial step of fatty acid β-oxidation. Its activity promotes melanoma cell survival and metastasis [17]
Aconitase 2	*ACO2*	Aconitate hydratase, mitochondrial. Catalyzes the isomerization of citrate to isocitrate via cis-aconitate in the TCA cycle. Enriched in melanoma small extracellular vesicles [18]
Acyl-CoA Thioesterase 1	*ACOT1*	Acyl-coenzyme A thioesterase 1, mitochondrial. Catalyzes the hydrolysis of acyl-CoAs, regulating intracellular levels of acyl-CoAs, free fatty acids, and coenzyme A. Associated with a lipogenic profile in uveal melanoma spheroids [19]
Acyl-CoA Thioesterase 13	*ACOT13*	Acyl-coenzyme A thioesterase 13, N-terminally processed, mitochondrial. High expression is associated with poor clinical outcomes in lung cancer [20]
Acyl-CoA Synthetase Long Chain Family Member 4	*ACSL4*	Long-chain-fatty-acid-CoA ligase 4, also mitochondrial. Catalyzes the conversion of long-chain fatty acids to acyl-CoA for both synthesis of cellular lipids and degradation via β-oxidation. Oxidative stress-related prognostic marker for melanoma metastasis [21]
Adenylate Kinase 3	*AK3*	GTP:AMP phosphotransferase AK3, mitochondrial. Involved in maintaining the homeostasis of cellular nucleotides by catalyzing the interconversion of nucleoside phosphates. AK3 knockout decreases proliferation and ATP levels in HeLa cells [22]
ATPase H+ Transporting V1 Subunit C1	*ATP6V1C1*	V-type proton ATPase subunit C 1; subunit of the peripheral V1 complex of vacuolar ATPase. V-ATPase is responsible for acidifying a variety of intracellular compartments in eukaryotic cells. ATP6V1C1 knockdown prevents breast cancer growth and bone metastasis [23]
Caspase 7	*CASP7*	Caspase-7 subunit p11. Involved in the activation cascade of caspases responsible for apoptosis execution. The caspase-7 inhibitor XIAP hampers melanoma invasion [24]
Catalase	*CAT*	Catalase. Protects cells from the toxic effects of hydrogen peroxide. Promotes melanoma growth. Inhibits ROS-induced cancer cell death [25]
CDGSH Iron Sulfur Domain 2	*CISD2*	CDGSH iron–sulfur domain-containing protein 2, also mitochondrial. Antagonizes BECN1-mediated cellular autophagy at the endoplasmic reticulum. CISD2 is aberrantly upregulated in malignant tumors [26]
Cleft Lip and Palate Transmembrane Protein 1-Like Protein	*CLPTM1L*	Cleft lip and palate transmembrane protein 1-like protein. TERT-CLPTM1 locus polymorphism is associated with melanoma risk [27]
COP9 Signalosome Subunit 3	*COPS3*	COP9 signalosome complex subunit 3. Component of the COP9 signalosome complex, an essential regulator of the ubiquitin conjugation pathway. The complex is also involved in phosphorylation of p53/TP53, c-jun/JUN, IκBα/NFKBIA, ITPK1, and IRF8/ICSBP. Acts as an oncogene in different cancers [28]
Cytochrome P450 Family 51 Subfamily A Member 1	*CYP51A1*	Lanosterol 14-alpha demethylase. A cytochrome P450 monooxygenase involved in sterol biosynthesis. Its inhibition decreases mitochondrial cholesterol and overcomes EGFR-TKI resistance in lung cancer cells [29]
Dermcidin	*DCD*	Survival-promoting peptide. DCD encodes the proteolysis-inducing factor core peptide (PIF-CP) and the skin antimicrobial peptide DCD-1. It may act as a pro-survival oncogene in various cancers, and it may represent a therapeutic target in melanoma [30]
G Elongation Factor Mitochondrial 1	*GFM1*	Elongation factor G, mitochondrial. Mitochondrial GTPase that catalyzes GTP-dependent ribosomal translocation during translation elongation. Associated with poor outcome in lung adenocarcinoma [31]
GON7 Subunit of KEOPS Complex	*GON7*	EKC/KEOPS complex subunit GON7, mitochondrial. Component of the tRNA-modifying EKC/KEOPS complex that represents a potential therapeutic target in TP53-mutated cancer cells [32]
H3 Clustered Histone 1	*HIST1H3A*	Histone H3.1. Core component of nucleosome. Recurrently mutated in diffuse intrinsic pontine gliomas [33]
Hexosaminidase Subunit Beta	*HEXB*	Hexosaminidase subunit beta chain A. Responsible for the degradation of GM2 gangliosides and other molecules containing terminal N-acetyl hexosamines. Hallmark of melanoma progression and poor survival [34]
Isocitrate Dehydrogenase (NADP(+)) 2	*IDH2*	Isocitrate dehydrogenase [NADP], mitochondrial. Plays a role in intermediary metabolism and energy production. Gain-of-function mutations drive tumor progression via D-2-hydroxyglutarate [35]
Isopentenyl-Diphosphate Delta Isomerase 1	*IDI1*	Isopentenyl-diphosphate Delta-isomerase 1. Involved in cholesterol synthesis. Upregulated following STAT6 silencing in lung cancer [36]
Keratin 86	*KRT86*	Keratin, type II cuticular Hb6. Knockdown of its KRT81 paralog inhibits melanoma progression [37]
LSM8 Homolog, U6 Small Nuclear RNA Associated	*LSM8*	U6 snRNA-associated Sm-like protein LSm8. Plays a role in pre-mRNA splicing as a component of the U4/U6-U5 tri-snRNP complex. Upregulated in various human cancers and unfavorable biomarker in 5-FU-treated gastric cancer patients [38]
Metaxin 2	*MTX2*	Metaxin-2, mitochondrial. Involved in the transport of proteins into the mitochondrion as part of the VDAC2/Mtx1/Mtx2 multi-protein complex that incorporates the mitochondrial pro-apotptic protein Bak [39]
NEDD8 Activating Enzyme E1 Subunit 1	*NAE1*	NEDD8-activating enzyme E1 regulatory subunit. Regulatory subunit of the dimeric UBA3-NAE1 E1 enzyme. Necessary for cell cycle progression through the S m checkpoint. Inhibition of the neddylation pathway represses cancer cell growth [40]
Nipsnap Homolog 2	*NIPSNAP2*	Protein NipSnap homolog 2, mitochondrial. Modulator of mitochondrial calcium channels, it participates in mitophagy [41]
Nitrilase Family Member 2	*NIT2*	Omega-amidase NIT2, mitochondrial. A nitrilase that converts α-ketoglutaramate and α-ketosuccinamate to α-ketoglutarate and oxaloacetate, respectively. Its downregulation inhibits colon cancer cell proliferation [42]
Nicotinamide Nucleotide Transhydrogenase	*NNT*	NAD(P) transhydrogenase, mitochondrial. The transhydrogenation between NADH and NADP is coupled to respiration and ATP hydrolysis and functions as a proton pump across the membrane. Its knockdown activates glucose catabolism in melanoma cells [43]
NPC Intracellular Cholesterol Transporter 2	*NPC2*	NPC intracellular cholesterol transporter 2. Involved in the egress of cholesterol from the lysosomal compartment. Highly expressed in vertical growth phase melanomas and lymph node metastases [44]
Phosphofructokinase, Muscle	*PFKM*	ATP-dependent 6-phosphofructokinase, muscle type. Catalyzes the phosphorylation of D-fructose-6-phosphate to fructose-1,6-bisphosphate by ATP, the first committing step of glycolysis. Participates in the metabolic rewiring in NRAS-mutated melanoma [45]
Peptidase, Mitochondrial Processing Subunit Beta	*PMPCB*	Mitochondrial-processing peptidase subunit beta. Catalytic subunit of the essential mitochondrial processing protease that cleaves the mitochondrial sequence off newly imported precursor proteins. Contributes to tumor cell resistance against sorafenib [46]
Paraoxonase 2	*PON2*	Serum paraoxonase/arylesterase 2. Hydrolyzes lactones and several aromatic carboxylic acid esters. Exerts a protective role against ROS production within the mitochondrial respiratory chain. Its expression correlates with melanoma progression [47]
Palmitoyl-Protein Thioesterase 1	*PPT1*	Palmitoyl-protein thioesterase 1. Removes thioester-linked fatty acyl groups from modified cysteine residues in proteins or peptides during lysosomal degradation. Promotes tumor growth and is associated with poor prognosis in various cancers, including melanoma [48]
Quinolinate Phosphoribosyltransferase	*QPRT*	Nicotinate-nucleotide pyrophosphorylase [carboxylating]. Involved in the catabolism of quinolinic acid in the kynurenine pathway and mitochondrial dynamics. Modulates progression, metastasis, and invasion of breast cancer [49]
Stearoyl-CoA Desaturase	*SCD*	Acyl-CoA desaturase, mitochondrial. Stearyl-CoA desaturase catalyzes the insertion of a *cis* double bond into fatty acyl-CoA substrates. Regulates mitochondrial fatty acid oxidation and is required for MITF-mediated melanoma cell proliferation [50]
Succinate Dehydrogenase Complex Flavoprotein Subunit A	*SDHA*	Succinate dehydrogenase [ubiquinone], mitochondrial. Flavoprotein subunit of succinate dehydrogenase that functionally couples the TCA cycle with the electron transfer associated with OxPhos. Loss-of-function mutations increase the propensity for cellular transformation and tumor development [51]
Succinate Dehydrogenase Complex Iron Sulfur Subunit B	*SDHB*	Succinate dehydrogenase [ubiquinone], mitochondrial. Iron–sulfur protein subunit of succinate dehydrogenase. See SDHA [51]
Solute Carrier Family 25 Member 1	*SLC25A1*	Tricarboxylate transport protein, mitochondrial. Citrate transporter that mediates the exchange of mitochondrial citrate for cytosolic malate. Plays a pro-oncogenic role and may represent a prognostic biomarker in different cancers [52]
SRY-Box Transcription Factor 5	*SOX5*	Transcription factor SOX-5. Binds specifically to the DNA sequence 5′-AACAAT-3′. Highly expressed in melanoma cells, it inhibits MITF expression and is involved in melanocyte differentiation [53]
Succinate-CoA Ligase ADP-Forming Subunit Beta	*SUCLA2*	Succinate--CoA ligase [ADP-forming] subunit beta, mitochondrial. ATP-specific succinyl-CoA synthetase functions in the TCA cycle, coupling the hydrolysis of succinyl-CoA to the synthesis of ATP. Its expression correlates with catalase levels and metastatic potential in lung and breast cancer patients [54]
Translin	*TSN*	Translin. DNA-binding protein that specifically recognizes consensus sequences at the breakpoint junctions in chromosomal translocations. Suppresses genome instability in Dicer-deficient cancers [55]
Ubiquitin Related Modifier 1	*URM1*	Ubiquitin-related modifier 1. Acts as a sulfur carrier required for 2-thiolation of various cytosolic tRNAs. Promotes tumor growth and suppresses apoptosis in hepatocellular carcinoma [56]

## Data Availability

The data presented in this study are available in the Supplementary Materials here.

## Supplementary Materials

The following supporting information can be downloaded at: https://www.mdpi.com/article/10.3390/ijms25053062/s1.

## Abbreviations

FDR: False Discovery Rate; GALC: β-galactosylceramidase; GEP, genome-wide expression profiling; GO: Gene Ontology; GSEA: Gene Set Enrichment Analysis; LC-MS/MS: Liquid Chromatography–Tandem Mass Spectrometry.
