# Supplementary material for: Proteomic Analysis Highlights the Impact of the Sphingolipid Metabolizing Enzyme β-Galactosylceramidase on Mitochondrial Plasticity in Human Melanoma
Source: Int J Mol Sci. 2024 Mar 6;25(5):3062. doi: 10.3390/ijms25053062 (PMC10931563; doi:10.3390/ijms25053062)
Supplement: Supplementary file 1 [file ijms-25-03062-s001.zip › Supplementary Figure S1.pdf]

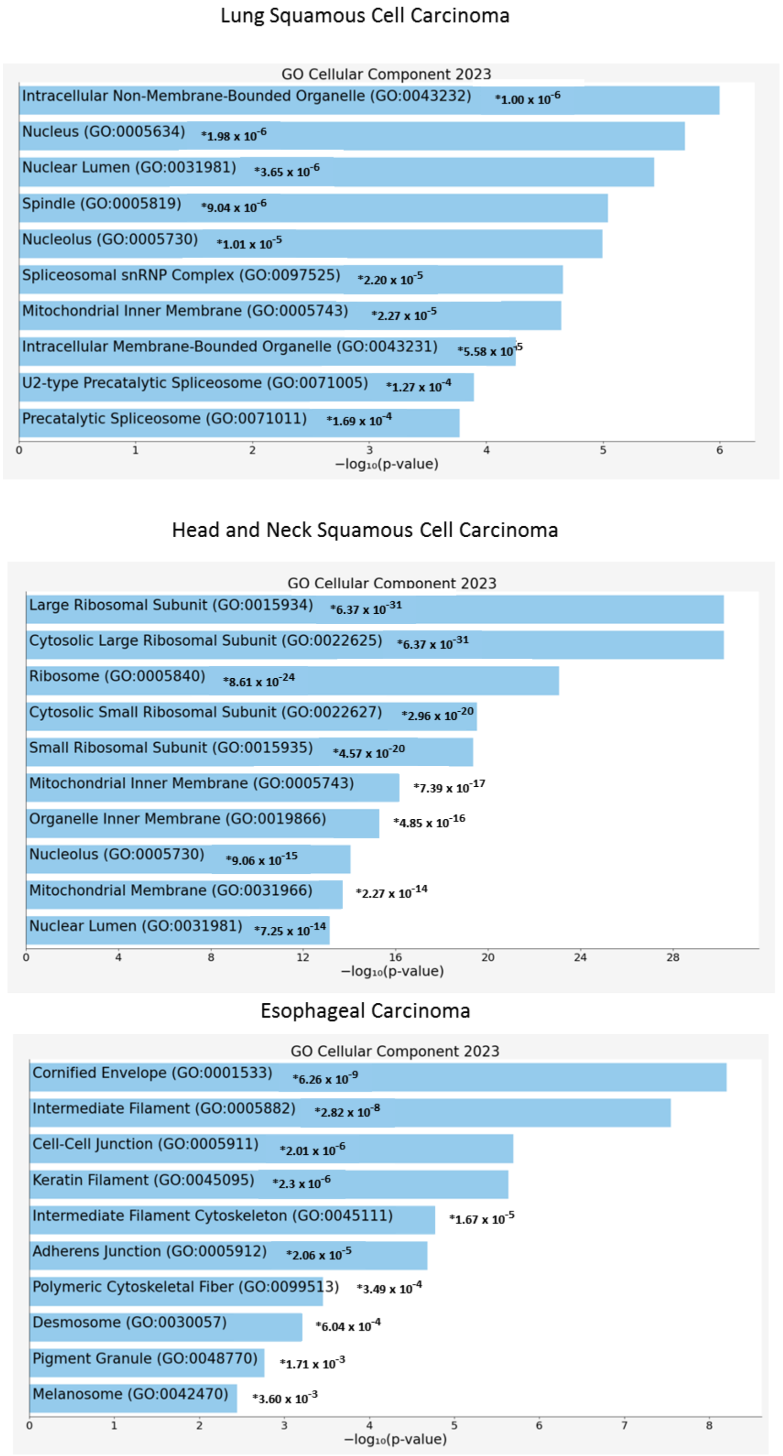

Supplementary Figure S1. GO categorization of the genes negatively correlated to *GALC* expression in human cancers. GO categorization was performed on the top 200 genes whose expression levels are negatively correlated with *GALC* expression in the indicated human tumors (TCGA, Firehose Legacy) following data mining on the cBioPortal for Cancer Genomics platform. At variance with the human tumors indicated in Figure 3, no correlation appears to exist among the selected genes and GO Cellular Component terms related to mitochondrial plasticity in these cancer types.
